# The influence of steroid type on outcomes in patients with acute respiratory distress syndrome

**DOI:** 10.1186/s40560-023-00681-4

**Published:** 2023-07-10

**Authors:** Shodai Yoshihro, Shunsuke Taito, Tomoaki Yatabe

**Affiliations:** 1 Department of Pharmaceutical Services, Hiroshima University Hospital, 1-2-3, Kasumi, Minami-Ku, Hiroshima, 734-8551 Japan; 2 Division of Rehabilitation, Department of Clinical Practice and Support, Hiroshima University Hospital, 1-2-3, Kasumi, Minami-Ku, Hiroshima, 734-8551 Japan; 3 Emergency Department, Nishichita General Hospital, 3-1, Nakanoike, Tokai-Shi, Aichi, 477-8522 Japan

**Keywords:** Acute respiratory distress syndrome, Dosing, Corticosteroid, Pharmacology

## Abstract

**Background:**

Recent systematic reviews and meta-analyses have suggested that low-dose steroids are effective in the treatment of acute respiratory distress syndrome (ARDS). Recent guidelines recommend the use of low-dose steroids instead of high-dose steroids. These systematic reviews were conducted based on the concept that the effect of steroids is constant regardless of their type. We discuss whether the type of steroid used influences the outcomes in patients with ARDS.

**Main body:**

From a pharmacological standpoint, methylprednisolone has little activity as a mineralocorticoid and may cause pulmonary hypertension. The results of the rank probability of our previous network meta-analysis revealed that low-dose methylprednisolone might be an optimal treatment compared to using other types of steroids or no steroids in terms of ventilator-free days. Similarly, an analysis of individual data from four randomized controlled trials suggested that low-dose methylprednisolone was associated with decreased mortality in patients with ARDS. Dexamethasone has attracted the attention of clinicians as a novel adjunct therapy for ARDS.

**Conclusion:**

Recent evidence has shown that low-dose methylprednisolone may be an effective treatment option for ARDS. The timing of initiation and duration of low-dose methylprednisolone therapy should be verified in future studies.

## Background

Steroids inhibit the progression of acute respiratory distress syndrome (ARDS) via an anti-inflammatory response. In ARDS, inflammation of the alveoli and microvessels worsens pulmonary edema. The collapse of alveolar tissue due to pulmonary edema can lead to filler formation following structural remodeling. Steroids control pulmonary edema by attenuating inflammatory cytokine levels.

Recently, the Japanese Society of Intensive Care Medicine published a revision of the ARDS Clinical Practice guidelines [1]. Although the previous version recommended against steroid administration, recent guidelines recommend the administration of low-dose steroids [1]. Recent evidence is based on the presumption that steroids have a “class effect” in patients with ARDS. For example, the Japanese ARDS practice guidelines define low-dose steroids as 1–2 mg/kg methylprednisolone equivalent dosages [1]. Similarly, previous systematic reviews included randomized controlled trials (RCTs) of various steroids, such as hydrocortisone, methylprednisolone, and dexamethasone [2–5]. However, the above-mentioned guidelines suggest the use of high-dose corticosteroids [1]. In addition, our network meta-analysis showed that low-dose methylprednisolone might be the optimal treatment, whereas using high-dose methylprednisolone or no steroids may be inferior to other treatments in terms of mortality, infection, and ventilator-free days (VFDs) [6]. Therefore, we discuss how the clinical efficacy of steroids in patients with ARDS may vary depending on the type of low-dose steroid used.

### Evidence to support the effects of steroids in patients with ARDS

Recently, positive evidence for the use of steroids in patients with ARDS has been established. As mentioned previously, the latest Japanese ARDS practice guidelines strongly suggest the use of low-dose steroids in adult patients with ARDS [1]. The recommendation was based on a systematic review of methylprednisolone, dexamethasone, and hydrocortisone, which were defined as “low-dose steroids”. Point estimates of the outcomes of interest also indicated a valid direction for low-dose steroids in the meta-analysis conducted in the guidelines. In a study comparing therapy with low-dose steroids to therapy without steroids, long-term mortality was reduced to 105 per 1000 patients, and infection was reduced to 50 per 1000 patients. In addition, the mean number of VFDs increased by 4.75 days, hospital stay decreased by a mean of 5.04 days, and ICU stay decreased by 5.23 days [1]. Similarly, a previous meta-analysis showed that systemic steroid therapy reduced mortality in patients with ARDS; however, this meta-analysis included a study on high-dose steroids [5].

Furthermore, this meta-analysis included an RCT that verified the effect of dexamethasone on ARDS caused by the coronavirus disease-2019 (COVID-19) [7]. The effect of steroids on viral ARDS was first reported in an RCT, although the pathogens were not reported in most eligible RCTs. Since interferon 1, which modulates the immune response to viral infections, was reported to be inhibited by dexamethasone and hydrocortisone in fundamental studies [8, 9], steroids were previously considered harmful to viral ARDS. However, this concept has evolved over time. Another systematic review suggested that the efficacy of systemic steroid therapy for COVID-19-related ARDS might be the same as that for traditional ARDS [4]. Therefore, the use of steroids might be approved in patients with ARDS, regardless of the cause.

### Type of steroids for patients with ARDS

**Fig. 1:**
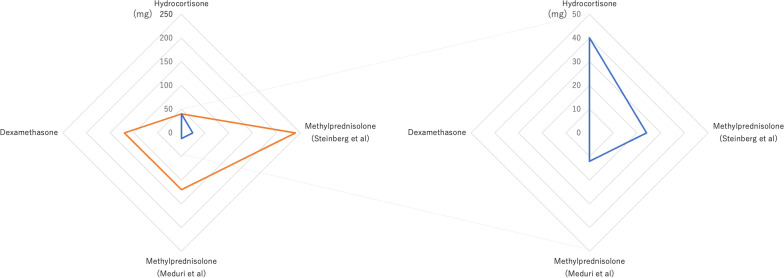
Radar chart of methylprednisolone equivalent dose for patients with acute respiratory distress syndrome weighing 60 kg on the initial day. The radar chart on the right is an enlarged view of a portion from the chart on the left. The orange line represents the activity of glucocorticoids, and the blue line represents the activity of mineralocorticoids. The steroid dose on the initial day (loading and maintenance doses) was based on the following randomized controlled trials: hydrocortisone [14], methylprednisolone (Steinberg et al.) [19], methylprednisolone (Meduri et al.) [20], and dexamethasone [17]. The potency of glucocorticoids and mineralocorticoids is shown as a methylprednisolone equivalent dose (mg per 60 kg body weight)

We propose that methylprednisolone is an effective steroid for the treatment of ARDS. The pharmacological effects of steroids are divided into those of glucocorticoids and mineralocorticoids, and their activities differ according to the type of steroid used (Fig. 1). Some in vitro studies have suggested that inhibiting the effect of mineralocorticoids may prevent the progression of pulmonary hypertension [10, 11]. An inappropriate increase in cultured pulmonary artery smooth muscle cells may suggest pulmonary vasculature remodeling, and mineralocorticoid receptors are associated with an increase in the number of cells [10]. Mineralocorticoid antagonists inhibited the elevation of the right ventricular systolic pressure in a mouse model of pulmonary hypertension [10]. Thus, mineralocorticoid agonists would deteriorate the pathology. Interstitial edema in ARDS compresses the pulmonary vasculature, which causes pulmonary vascular remodeling and pulmonary hypertension [12]. Considering the pathology of ARDS [12], the effect of mineralocorticoids may be an obstacle to the management of ARDS. The mineralocorticoid activities of methylprednisolone and dexamethasone were lower than that of hydrocortisone (Fig. 1). A recent systematic review on community-acquired pneumonia indicated that steroids were not associated with improved mortality in patients, similar to a sub-analysis by a group administered steroids to the ICU [13]. In this systematic review, of the 16 eligible studies, five administered hydrocortisone and three administered methylprednisolone as an intervention [13]. The type of steroids used may have affected the results of this systematic review. Additionally, an RCT using hydrocortisone reported negative clinical outcomes in patients with ARDS [14]. A recent analysis using 18,106 of Korean health insurance claims data showed that hydrocortisone was not associated with a decrease in 180-day mortality in patients with non-viral and COVID-19-related ARDS [15]. Our network meta-analysis also showed that hydrocortisone might be inferior to low-dose methylprednisolone and dexamethasone in terms of VFDs (Table 1) [6]. Another analysis of individual data from four RCTs suggested that low-dose methylprednisolone improved mortality in patients with ARDS [16].

**Table 1:** Balance of benefits and herms among each steroid

	Mortality		Infection		VFD	
	Absolute effect	Rank	Absolute effect	Rank	Absolute effect	Rank
High-dose methylprednisolone	33/1000 fewer (486 fewer–296 more)	4	61/1000 fewer (52 fewer–337 more)	5		
Low-dose methylprednisolone	138/1000 fewer (290 fewer–94 more)	1	131/1000 fewer (240 fewer–23 more)	1	MD 6.06 higher (2.5 higher–10.5 higher)	1
Hydrocortisone	138/1000 fewer (362 fewer–198 more)	2	2/1000 more (95 fewer–169 more)	3	MD 2.10 higher (3.15 lower–7.09 higher)	3
Dexamethasone	71/1000 fewer (305 fewer–262 more)	3	50/1000 fewer (139 fewer–60 more)	2	MD 3.64 higher (0.56 lower–7.83 higher)	2
No steroid	–	5	–	4	–	4

No steroid: using no steroid, MD: mean difference, (): 95% 95% confidence interval

We made this original table base on results of our previous study [6]

Dexamethasone has come to the forefront as an adjunct therapy for ARDS. One RCT reported that dexamethasone improved 60-day mortality in patients with ARDS [17]. Another RCT reported that dexamethasone increased VFDs in patients with COVID-19-related ARDS [7]. Similarly, an analysis of Korean health insurance claims data showed that dexamethasone was associated with a decrease in the 180-day mortality in patients with non-viral and COVID-19-related ARDS [15]. The risk bias of these RCTs was high because a placebo was not used in the control group, and the design of the analysis using health insurance claims data was not an RCT. In contrast, a recent small RCT, although not targeting only patients with ARDS, directly compared 1 mg/kg/day of methylprednisolone for 7 days with 8 mg/day dexamethasone for 7 days in patients with COVID-19 administered in the ICU [18]. An RCT report indicated that dexamethasone reduced mortality and increased secondary infections [18]. In addition, our network meta-analysis indicated that dexamethasone may be inferior to low-dose methylprednisolone and hydrocortisone in terms of the mortality rate (Table 1) [6]. The in vitro study suggests that both excessive suppression of cytokines and high cytokine levels promote bacterial growth [19]. This implies that the relationship between the dosage of steroids and the incidence of infection in patients with ARDS could be U-shaped. A previous study suggested that methylprednisolone may have a higher likelihood of migrating to the lungs, liver, and muscle compared to dexamethasone in a rat model [20]. While no studies examined the levels of cytokine in each organ, the differences in tissue migration may account for the varying incidence of infection in patients with ARDS. Therefore, methylprednisolone should be selected as a steroid in patients with ARDS. The results of each point estimate and ranking in our network meta-analysis showed differences between the types of steroids.

### Future studies for steroids in patients with ARDS

As shown in Table 2, steroid therapy for ARDS in RCTs [7, 14, 17, 21, 22] was heterogeneous not only in type, but also in dosage, period, and initiation, which was referred to in the systematic review by Hirano et al. [3].

**Table 2:** Details of studies for patients with ARDS

Author year	No of patients steroid/control	Main etiology	Initiation timing of steroids after the diagnosis of ARDS	Regimen of steroids	Comparison	Planned primary outcome
Steinberg 2006 [18]	89/91	Pneumonia 42% (76/180) Sepsis 22% (40/180)	7–24 days	Methylprednisolone Loading: 2 mg/kg Day1–14: 0.5 mg 4 times daily Day15–21: 0.5 mg 2 times daily Tapering off over 4 days	Placebo (5% dextrose)	Mortality at day 60
Meduri 2007 [19]	63/28	Pneumonia 42% (38/91) Sepsis 16% (15/91)	Less than 72 h	Methylprednisolone Loading: 1 mg/kg Day1–14: 1 Day15–21: 0.5 Day22–25: 0.25 Day26–28: 0.125 mg/kg/day by continuous infusions	Placebo (0.9% serine)	1-point reduction in lung injury score
Tongyoo 2016 [13]	98/99	Pneumonia 51% (100/197)	Less than 12 h	Hydrocortisone Day1–7: 50 mg 6 times daily	Placebo (Not detail)	No
Tomazini 2020 [4]	151/148	Pneumonia due to COVID-19	Less than 24 h	Dexamethasone Day1–5: 20 Day6–10: 10 mg once daily	No placebo	Ventilator-free days to day 28
Villar 2020 [16]	139/138	Pneumonia 53% (147/277) Sepsis 24% (67/277)	Less than 24 h	Dexamethasone Day1–5: 20 Day6–10: 10 mg once daily	No placebo	Ventilator-free days to day 28
Edalatifard 2020 [21]	34/28	Pneumonia due to COVID-19	24–48 h	Methylprednisolone 250 mg 3 days	No placebo	Radiographic findings Mortality (Not detail of observational period), etc.

*ARDS* acute respiratory syndrome, *COVID-19* coronavirus disease 2019

Notably, the methylprednisolone dose should be low. From past studies, we suggest that “low-dose” methylprednisolone was the initial dosage in RCTs by Meduri et al. and Steinberg et al., that is, each initial dosage was 1 or 2 mg/kg for loading and 1 or 2 mg/day for maintenance [21, 22]. The efficacy of this dosage was supported by the above-mentioned studies [6, 16]. However, high-dose methylprednisolone as an adjunctive therapy for ARDS may be harmful, similar to pulsed methylprednisolone therapy. The aforementioned guidelines recommend against the administration of 30 mg/kg methylprednisolone as an initial dose [1]. A multicenter propensity-matched cohort study suggested that pulsed methylprednisolone therapy is associated with an increase in 60-day mortality [23]. One RCT using 250 mg/day methylprednisolone for 3 days reported improved mortality in COVID-19-related ARDS [24]. However, the permissible increase in methylprednisolone dose is unknown. Considering that the optimal timing of initiation and duration of steroid therapy is also under discussion, steroids need to be reviewed as an adjunctive therapy for ARDS to design tailor-made medication therapies based on patient characteristics.

## Conclusion

Steroid therapy for patients with ARDS is effective, as supported by a recent meta-analysis. Low-dose methylprednisolone may be the most effective steroid for ARDS. The timing of initiation and duration of low-dose methylprednisolone therapy should be verified in future studies.

## Data Availability

Not applicable.

## Abbreviations

ARDS: Acute respiratory syndrome
COVID-19: Coronavirus disease 2019
RCT: Randomized control trial
VFDs: Ventilator-free days
